# Genomic Epidemiology of Foodborne *Salmonella* in Colombia (2002–2009): Emergence of Novel IncHI1 and IncI1 Plasmids Harboring Metal and Multi-Drug Resistance Clusters

**DOI:** 10.3390/antibiotics15050511

**Published:** 2026-05-18

**Authors:** Menghan Li, Guerrino Macori, Salim Mattar, Li Bai, Séamus Fanning

**Affiliations:** 1 National Health Commission Key Laboratory of Food Safety Risk Assessment, China National Center for Food Safety Risk Assessment, Beijing 100021, China; limenghan@cfsa.net.cn; 2 UCD Centre for Food Safety, School of Public Health, Physiotherapy & Sports Science, University College Dublin, D04 N2E5 Dublin, Ireland; 3 UCD Centre for Food Safety, School of Biology & Environmental Science, University College Dublin, D04 N2E5 Dublin, Ireland; guerrino.macori@ucd.ie; 4 Institute for Tropical Biological Research, School of Veterinary Medicine, University of Cordoba, Montería 230002, Colombia; smattar@correo.unicordoba.edu.co

**Keywords:** *Salmonella*, whole-genome sequencing (WGS), antimicrobial resistance (AMR), IncHI1 plasmid, CHASRI, foodborne pathogens, genomic epidemiology

## Abstract

**Background/Objectives:** Multidrug resistant (MDR) *Salmonella* represents a major global public health challenge within the One Health interface. This study aimed to characterize the genomic epidemiology of *Salmonella* isolates from Colombia and resolve the genetic architecture of novel MDR plasmids identified in foodborne strains. **Methods:** A total of 90 *Salmonella* isolates collected between 2002 and 2009 from various food sources and food-producing animals in Colombia were analyzed using whole-genome sequencing (WGS). Bioinformatics tools were employed for serotype prediction, multi-locus sequence typing (MLST), and resistome/virulome profiling. Long-read sequencing was utilized to close the complete sequences of representative MDR plasmids. **Results:** 45.6% of isolates exhibited antimicrobial resistance, with seven being classified as MDR. The major serotypes identified were Uganda (n = 20), Newport (n = 10), and Braenderup (n = 10). We characterized a novel 229,037 bp IncHI1 plasmid (pCFS0255-1) harboring a copper homeostasis and silver resistance island (CHASRI) integrated with tetracycline and macrolide resistance clusters. Additionally, a 99,288 bp IncI1 plasmid (pCFS0255-2) carrying a unique aminoglycoside resistance module was resolved. **Conclusions:** Our findings highlight the persistence of specific *Salmonella* lineages in the Colombian food chain and the role of hybrid plasmids in the co-selection of metal and antibiotic resistance. The study underscores the necessity of implementing WGS-based surveillance to track emerging MDR threats.

## 1. Introduction

*Salmonella*, a Gram-negative bacterium, is one of the most widely known foodborne pathogens, often involved in food safety incidents worldwide and which poses a challenge to public health, especially in developing countries [1].

Antimicrobial resistance (AMR) is a major global societal threat of growing concern to One Health. It also has an impact on food security as well as the economic well-being of millions of people. Among other zoonotic infectious diseases, antibiotic-resistant *Salmonella* in food chains associated with livestock are of concern [2]. Mobile genetic elements (MGE) such as plasmids, transposons, and insertion sequence elements can mediate the spread of not only AMR genes but also heavy metal- and biocidal resistance-encoding genes, thus limiting the treatment and control of *Salmonella* [3].

Whole-genome sequencing is more widely used in foodborne pathogen risk assessment [4]. It enables a variety of diagnostic applications using a streamlined pipeline, including pathogen subtyping, source tracking, and virulence gene identification, along with prediction of AMR genotypes [5].

Due to the existence of transnational food trade, the evolution of foodborne disease threats requires cross-border coordination to ensure that local food safety risks are controlled before they become international issues [6]. Within the One Health framework, some countries and regions have attempted to establish molecular tracing networks for foodborne pathogens. For example, the GenomeTrakr tracing network, led by the U.S. FDA (Food and Drug Administration), was initiated in 2012 and has since developed into a WGS (whole-genome sequencing)-based regulatory network that includes cooperation from 40 U.S. states and 30 international laboratories [7]. In China, the National Foodborne Disease Surveillance Molecular Tracing Network (TraNet) was initiated in 2013 to conduct precise tracing of various foodborne pathogens, including *Salmonella*, using both PFGE (Pulsed-Field Gel Electrophoresis) technology and cgMLST (core-genome multi-locus sequence typing)-based WGS classification [8,9]. In 2021, the ECDC (European Centre for Disease Prevention and Control) introduced the EpiPulse online collaboration framework, aiming to integrate the foodborne disease surveillance networks of European countries to strengthen cross-border tracing cooperation [10]. These molecular tracing networks have effectively established a nationwide and cross-border surveillance system across the collaboration public health laboratories. However, the threat of foodborne diseases may originate from regions and pathogens that are not yet included in surveillance networks. The cgMLST tracing method provides stable allele-based typing, but interpretation depends on the availability of contextual genomic datasets, presenting certain limitations when identifying strains that belong to newly emerging pathogenic species or those that are distantly related to known types. For countries or regions not included in the regulatory network, the inability to submit WGS data for centralized monitoring and analysis further limits the effectiveness of cgMLST-based tracing methods. Without access to centralized surveillance networks, these areas face challenges in identifying and classifying strains accurately through cgMLST.

Therefore, it is essential to develop a standardized WGS tracing and analysis workflow that does not rely on existing databases to address emerging and unknown food safety risks, particularly those originating from regions not connected to regulatory networks or involving pathogens that are not included in current databases.

In this study, we developed a strain tracing workflow using core-genome SNPs for phylogenetic analysis, which includes the identification of antimicrobial resistance (AMR) genes and *Salmonella* pathogenicity islands (SPIs). Based on this workflow, WGS was applied to 90 *Salmonella* species cultured from sources representing the One Health continuum in Colombia. Genetic relationships among these bacterial isolates were investigated, as well as their AMR genotypes. Specifically, for a tetracycline–aminoglycoside–gentamicin multidrug resistance (MDR) module identified, long-read sequencing was performed to close the genome and analyze the nature of these MDR plasmids.

## 2. Results

### 2.1. Geno- and Phenotypic Characterization of the 90 Salmonella Collection

#### 2.1.1. Metadata, AST Phenotype, Serotyping, and Phylogenetic Tree

A total of 90 *Salmonella* isolates were cultured from food and food-producing and other animals in Colombia between 2002 and 2009. Majority of these sources included meat such as pork, chicken, beef, and their products. Non-meat products such as flour mandioka powder and a mixture of corn and egg were also included. Exotic animal sources included *Iguana iguana*, *Hydrochoerus hydrochaeris,* and *Trachemys scripta callirostris*.

AST results for a panel of 12 antibiotics that were tested demonstrated that there are 41 AMR-expressing isolates, including seven classified as multidrug resistant (MDR), among the collection. In addition, except for nine completely susceptible isolates, all other isolates expressed an intermediate resistance phenotype, as determined by the disk diffusion method used.

**Figure 1:**
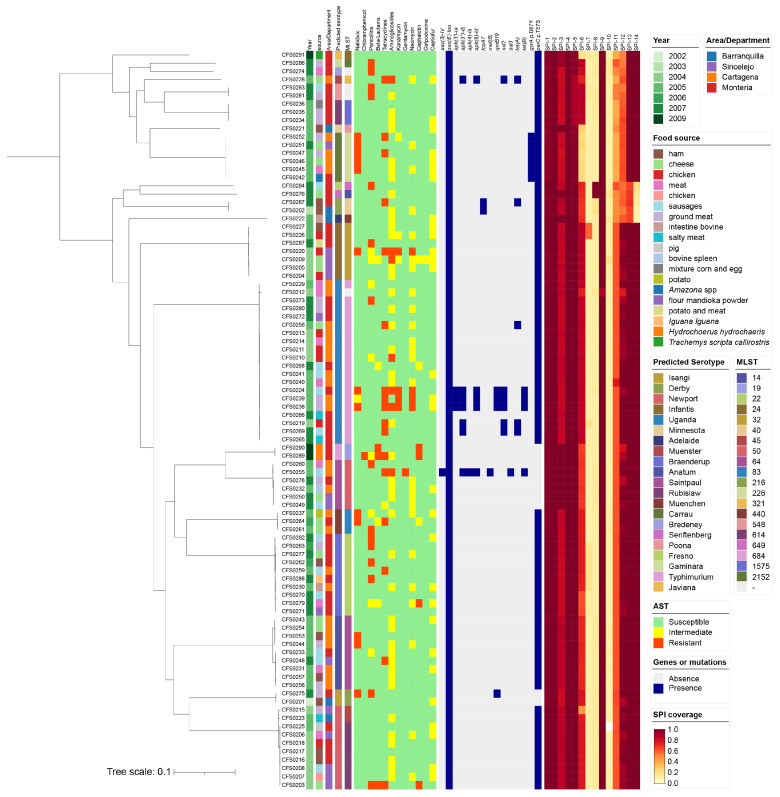
Source information, phenotype, and genotype of 90 *Salmonella* isolates from Colombia. Information from the metadata included year of isolation, food source, geographical region/department, AST result, and presence/absence of resistance genes or site mutations. Gradient colors are used to represent SPI coverage.

The serotype prediction results based on the whole-genome sequence indicated that there were 21 serotypes identified, of which Uganda (n = 20), Newport (n = 10), Braenderup (n = 10) and Anatum (n = 9) were in the majorities. The predicted results of the in silico Multi-locus sequence typing (MLST) corresponded with the presentation of serotypes, as shown in Figure 1. We observed discrepancies in the predicted serotypes from WGS and the laboratory-determined serotypes for 20 isolates. The detailed comparison is provided in the metadata table (Appendix A Table A1).

Comparative genomic analysis of the 90 *Salmonella* isolates identified a total of 3274 core genes. The recombination-filtered core genome alignment spanned 3,126,423 bp. From this alignment, a total of 110,546 informative SNPs were extracted to construct a high-resolution maximum-likelihood phylogenetic tree.

#### 2.1.2. AMR Genotypes in Isolates of the Collection

In silico identification of AMR genes and point mutations indicated that some individual isolates had several different antibiotic resistance-encoding genes representing different drug classes, while *aac(6′)Iaa* was detected in all 90 isolates. CFS0228 had a relatively unique *aph(3″)Ib-sul2-tet(A)-aph(6)Id* AMR genotype module, and this corresponded to the phenotype aminoglycoside, sulfonamide, and tetracycline resistance, with intermediate resistance to ceftiofur and neomycin.

CFS0129 and CFS0269 were determined to have similar AMR genotypes to CFS0228, except for *aph(6)Id*. However, their resistance phenotype was relatively simple, expressing resistance to tetracycline only. The lack of sulfonamide and aminoglycoside resistance, despite the presence of *sul2* and *aph(3″)Ib*, suggests that these genes might be non-functional. The years in which these two were recovered were quite different. CFS0219 was isolated in 2004 from chicken and CFS0269 in 2007 from sausage.

CFS0224, CFS0238, and CFS0239 had the same AMR genotype: *aph(3″)Ib-sul2-aph(6)Id-aph(3′)Ia-qnrB19-tet(B)*. At the same time, they expressed a similar resistance phenotype to kanamycin, neomycin, and tetracycline. CFS0224 and CFS0238 also expressed resistance to aminoglycosides and nalidixic acid, while CFS0238 and CFS0239 was intermediately resistant to ceftiofur. These three isolates were all recovered in 2005. CFS0238 and CFS0239 were cultured from ground meat samples, and CFS0224 was recovered from sausage. Further, the three isolates were same serotype and MLST profile, and were very close in evolutionary distance, although CFS0224 was derived from Monteria rather than Cartagena, the location for the other two isolates.

CFS0255 expressed a novel resistance genotype: *tet(B)-aph(3″)Ib-aph(6)Id-aph(4)Ia-aac(3)IV-mef(B)-sul3*, corresponding with its MDR phenotype aminoglycoside, gentamicin, and tetracycline.

#### 2.1.3. SPI Genotypes Identified in the Isolate Collection

The study collection was also assessed for the presence of *Salmonella* Genomic Islands (SGI) by in silico analysis. Among all the 90 isolates, SPI-1, SPI-2, SPI-3, SPI-4, SPI-5, and SPI-9 were determined to have a high coverage rate (>80%). SPI-6 has partial coverage in all isolates except Javiana, Minnesota, and Carrau serotypes. Similarly, SPI-7 was detected in CFS0226 and CFS0227 only, and were identified as serovar Infantis. SPI-8 was identified in CFS0276 and CFS0284, which were identified as the Fresno and Senftenberg serotypes, respectively. SPI-11 to SPI-14 were partially found in most isolates, while isolates CFS0202, CFS0222, CFS0267, CFS0276, and CFS0284 lacked SPI-14. SPI-10 was absent in all isolates, as shown in Figure 1.

### 2.2. Comparative Genomic Analysis of AMR Gene-Encoding Regions in WGS Assemblies

To better understand the distribution of AMR genes together with the upstream and downstream sequences, each of the AMR gene sequences in contigs mentioned above were studied to describe the nature of their AMR-encoding loci in addition to their upstream and downstream loci (to 5 kbp respectively).

Reference sequences from the NCBI NR/NT database of AMR regions with high identity and coverage, obtained via online BLASTN searches, are listed in Table 1.

**Figure 2:**
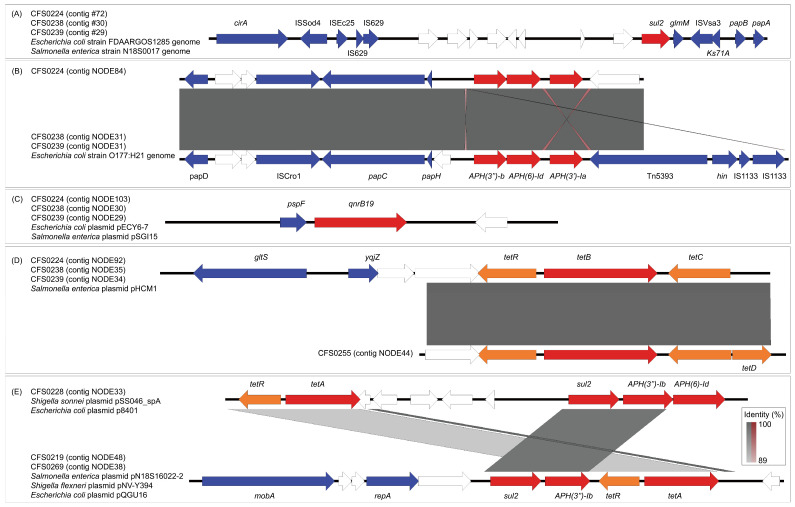
Genetic structures of the antibiotic resistance genes harboring contigs and comparisons against reference sequences. CDSs are shown as arrows with different colors, wherein AMR genes were colored red, AMR-associated genes were colored orange, and other known genes in blue. Blank arrows represent hypothetical genes. Shaded areas between each of two sequences are represented by direct (gray) or inverted (red) nucleotide identity regions between loci. Isolates and contig number are listed with related reference sequences. (**A**) Genetic structure of *sul2* flanking regions. (**B**) Genetic structure of *aph(3″)Ib-aph(6)Id-aph(3)Ia* flanking regions. (**C**) Genetic structure of *qnrB19* flanking regions. (**D**) Genetic structure of *tetB* flanking regions. (**E**) Genetic structure of *sul2-aph(3″)Ib-tetR-tetA* flanking region in CFS0219 and CFS0269, compared with *tetR-tetA-sul2-aph(3″)Ib-aph(6)Id* flanking region in CFS0228.

The pattern of distribution of antibiotic resistance genes was found to be highly consistent among isolates CFS0224, CFS0238, and CFS0239 (Figure 2A). The *sul2* resistance gene, the *qnrB19* resistance gene, the *tetR-tetB-tetC* cluster, and the *aph(3″)Ib-aph(6)Id-aph(3)Ia* resistance gene modules were located on four different contigs respectively. These contigs also shared high similarities among the three isolates. Sequence comparison showed that the contigs containing *sul2* were highly homologous to the reference sequences from the chromosomes of *Escherichia coli* FDAARGOS_1285 and *Salmonella enterica* subsp. enterica serovar Agona CVM N18S0017. Gene annotation indicated that there were multiple mobile genetic elements. *ISSod4*, *ISEc25*, *IS629*, and *ISVsa3* insertion sequences were found upstream and downstream of *sul2*.

Meanwhile, the contigs containing the *aph(3″)Ib-aph(6)Id-aph(3)Ia* gene cluster were highly identical to part of the chromosome of *Escherichia coli* O177:H21 (Figure 2B). In the upstream and downstream sequences of the *aph(3″)Ib-aph(6)Id-aph(3)Ia* module, a transposon Tn5393 and two copies of its related insertion sequence *IS1333* were identified.

The contigs harboring qnrB19 (Figure 2C) were found to be identical with the *Escherichia coli* plasmid pECY6-7, which is a 2.7-kbp ColE-like small plasmid with only three CDSs. The plasmid was also found in *Salmonella*, and designated plasmid pSGI15.

In isolates CFS0224, CFS0238, and CFS0239, we observed a tetracycline resistance gene cluster, *tetRBC*, which is highly homologous to the *Salmonella* plasmid pHCM1 (Figure 2D). In the contigs from the second-generation sequencing of CFS0255, we identified another part of this gene cluster. Although it does not match the upstream region of the initial cluster, it contains a more complete tetracycline resistance gene cluster, *tetRBCD*. This contig in CFS0255 was later confirmed to be a partial plasmid pCFS0255-1 through long-read sequencing analysis.

Isolate CFS0219 and CFS0269 each had a contig with the *sul2-aph(3″)Ib-tetR-tetA* module (Figure 2E). This region was identical to one located on three plasmids from different bacteria, including *Salmonella* plasmid pN18S1602-2, *Shigella flexneri* plasmid pNV-Y394, and *Escherichia coli* plasmid pQGU16. The flanking regions in this case are different when compared with one identified in the CFS0228 *tetR-tetA-sul2-aph(3″)Ib-aph(6)Id* gene cluster, although they share the same four resistance genes, while the latter mentioned is identical with referenced *Shigella sonnei* plasmid pSS046_spA and *Escherichia coli* strain plasmid p8401.

### 2.3. Genetic Synteny and Sequence Comparison of a Novel Multidrug Resistance IncHI1 Plasmid pCFS0255-1

Two plasmids were identified in the MDR isolate CFS0255 complete genome sequence. Results of the long- and short-read sequencing and hybrid assembly confirmed these as closed circular DNA sequences. Plasmid pCFS0255-1 is 229,037 bp in length, with 46.53% in GC content, while plasmid pCFS0255-2 is 99,288 bp in length, with 50.38% in GC content.

**Figure 3:**
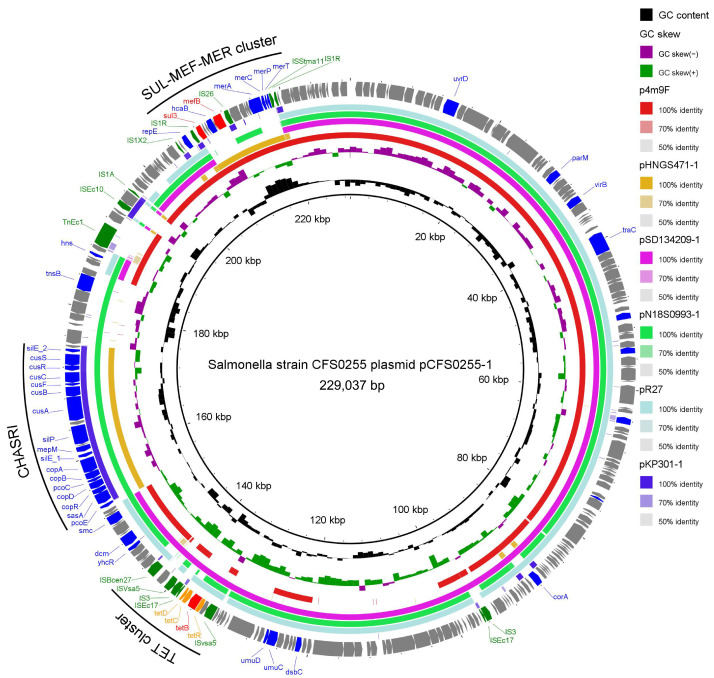
Circular schematic map of the novel multidrug resistance IncH1-like plasmid pCFS0255-1, showing the genetic structure of the plasmid. CDSs were shown as arrows. ARG, regulation genes of ARG genes, and insertion sequences were highlighted in green. Other annotated genes were denoted by blue colors. Colored arcs are used to show regions of homology between the plasmid sequence and its four reference sequences, including plasmid pHNGS471-1, plasmid pSD134209-1, plasmid pN18S0993-1, and plasmid R27. The inner two rings represented GC Skew [(G − C)/(G + C), G and C represents guanine content cytosine content] and GC content. Regions of the three antibiotic resistance and metal tolerance gene clusters were identified, including the TET cluster, the CHASRI, and the SUL-MEF-MER cluster.

Results obtained from annotation of these episomes using PlasmidFinder indicated that plasmid pCFS0255-1 was a IncHI1-like plasmid (Table 2). The genetic backbone structure of plasmid pCFS0255-1 was found to be highly identical to several reference plasmid complete genome sequences in NCBI GenBank database, including *Escherichia coli* 4M9F plasmid p4M9F (accession number MN256759.1) [11], *Klebsiella pneumoniae* GD21SC417 plasmid pHNGS471-1 (accession number: CP089510), *Escherichia coli* SD134209 plasmid pSD134209-1 (accession number: CP029690), *Salmonella enterica* strain CVM N18S0993 plasmid pN18S0993-1 (accession number: CP082572), *Salmonella* Typhi plasmid R27 (accession number: NC_002305), and *Klebsiella pneumoniae* strain CRE301 plasmid pKP301-1 (accession number: CP166314) [12]. Three resistance gene clusters were noted on plasmid pCFS0255-1, including the tetracycline resistance gene cluster (TET cluster), the copper homeostasis and silver resistance island (CHASRI), and the sulfonamide–macrolide–mercury resistance gene cluster (SUL-MEF-MER cluster) [13]. No other plasmids in the reference database were found containing all of these three gene islands mapped to one complete episome. Meanwhile, in the flanking region of each of the resistance gene islands, multiple copies of insertion sequences were found (Figure 3).

### 2.4. Genetic Synteny and Sequence Comparison of a Novel Aminoglycoside Resistance IncI1 Family Plasmid pCFS0255-2

**Figure 4:**
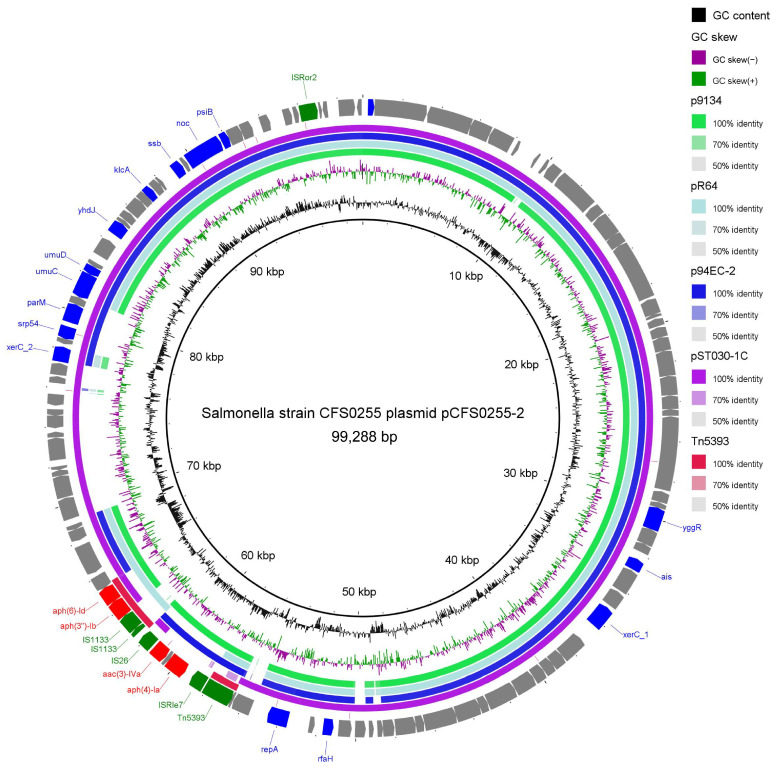
Circular schematic map of the novel multidrug resistance IncI1 family plasmid pCFS0255-2, displaying the genetic structure of the plasmid. CDSs were shown as arrows. Antibiotic resistance genes are highlighted in red. Insertion sequences were highlighted in green. Other annotated genes were denoted by a blue color. Colored arcs are used to show regions of homology between the plasmid sequence and its four reference sequences, including plasmid p9134, plasmid R64, plasmid p94EC-2, and transposon Tn5393. The inner two rings represent GC Skew and GC content.

Plasmid annotation of plasmid pCFS0255-2 suggested that it is a IncI-type plasmid. The basic backbone is highly identical to *Salmonella* Typhimurium 9134 plasmid p9134 (accession Number: KF705205.1) [14], *Salmonella* Typhimurium plasmid R64 (accession number: AP005147.1) [15], *Escherichia coli* 94EC plasmid p94EC-2 (accession number: CP047578.1) [16], and *Salmonella* Typhimurium plasmid pST1030-1C (accession number: MT507879.1) [17]. Plasmid pCFS0255-2 harbors an *aph(6)Id-aph(3″)Ib-aac(3)IVa-aph(4)Ia* aminoglycoside resistance-containing module, interspersed with insertion sequences *IS1133*, *ISRle7*, and *IS26*, which is not repeated in its entirety in the reference plasmid sequences. Smaller parts of the resistance gene cluster were found to be identical to a streptomycin resistance transposon, Tn5393 (accession number: M96392.1), described in 1993 in *Erwinia amylovora* [18]. All of the four reference plasmid sequences above contain a tetracycline resistance gene cluster, which is absent in plasmid pCFS0255-2 (Figure 4).

## 3. Discussion

### 3.1. Superiority of WGS for Serotyping Historical Isolates

The failure of traditional phenotypic serotyping (e.g., slide agglutination) in these older isolates can likely be attributed to several well-documented biological and technical limitations. These include the loss of surface antigens (such as the flagellar H-antigen) during prolonged storage, the transition to rough colony variants (resulting in the loss of the O-antigen), weak or non-specific auto-agglutination, and the potential use of incomplete antisera panels at the time of historical testing [19]. In contrast, in silico serotyping directly targets the underlying genetic determinants, providing a highly robust and objective prediction [20]. Importantly, the WGS-predicted serotypes are completely consistent with the clustering observed in the core-genome SNP phylogenetic tree, further confirming the superior accuracy of the genomic approach over historical laboratory records. This robust in silico confirmation establishes a reliable foundation for the subsequent genomic epidemiological analyses.

### 3.2. Evolutionary Insights and Mosaic Architecture of the MDR Plasmid pCFS0255-1

Comparative genomic analysis using the NCBI NR/NT database revealed that pCFS0255-1 possesses a unique assemblage of three resistance gene islands—TET, CHASRI, and SUL-MEF-MER—a combination not previously reported in a single closed plasmid sequence. Detailed synteny analysis with closely related replicons highlights the mosaic nature of this episome. While *Salmonella enterica* plasmid pN18S0993-1 shares high homology in the TET and CHASRI regions, it lacks the SUL-MEF-MER cluster. Conversely, plasmids from other genera, such as *Klebsiella pneumoniae* (pHNGS471-1) and *Escherichia coli* (p4M9F), harbor the SUL-MEF-MER module but diverge significantly in their backbone or other accessory repertoires. Interestingly, the pCFS0255-1 backbone exhibits the highest structural similarity to *Escherichia coli* plasmid pSD134209-1 and the ancestral *S.* Typhi R27 plasmid, yet these lack both the metal resistance and the broader antimicrobial resistance islands.

Notably, a specific subsequence (positions 194,016–196,464) flanked by insertion sequences *ISEc10* and *IS1A* was uniquely identified in *K. pneumoniae* plasmids (e.g., pKP301-1), suggesting a recent acquisition from this genus. Collectively, these findings support a hypothesis of modular evolution through multiple recombination events. It is plausible that these mobile genetic elements (MGEs) have facilitated the integration of diverse resistance determinants from across the Enterobacteriaceae family, including *Escherichia*, *Klebsiella*, and *Salmonella*. Such interspecies horizontal gene transfer (HGT) underscores the emergence of complex “super-plasmids” in the food production chain, presenting a fortified vehicle for the co-dissemination of multidrug and heavy metal resistance.

To address whether the modular structure of pCFS0255-1 represents a persistently circulating threat, we queried its complete sequence against the NCBI *nt_core* database (version 20250319). Notably, filtering for high homology (coverage ≥ 80%, weighted ANI ≥ 95%) yielded multiple highly similar plasmid records isolated predominantly from recent years (2013–2023). These plasmids were identified across a global geographic distribution, including the USA, China, South Korea, Thailand, Japan, and Vietnam (Supplementary Materials Table S1). However, the complete genetic architecture of this MDR plasmid has not been fully explored or discussed until now.

Furthermore, consistent with the One Health paradigm, these structurally conserved plasmids are actively circulating among diverse hosts and environments, including food-producing animals (swine and poultry), retail meats, and human clinical fecal samples. Notably, the backbone was also identified in other Enterobacteriaceae species, such as *Escherichia coli* (e.g., CP064017.1, isolated from pigs in Thailand, 2018) and *Klebsiella pneumoniae* (e.g., CP072461.1, isolated from swine in China, 2019). The extensive recent prevalence of this plasmid architecture globally underscores that the historical pCFS0255-1 identified in our 2005 Colombian isolate is not an evolutionary dead-end. Instead, it serves as a crucial historical baseline to track the long-term persistence, global dissemination, and cross-species horizontal transfer of this MDR ‘super-plasmid’ over nearly two decades.

### 3.3. The CHASRI Gene Cluster

The copper homeostasis and silver resistance island (CHASRI) was first described in 2016 [15]. This 19-gene metal resistance cluster is believed to have originated in *Enterobacter cloacae,* and has spread across the Enterobacteriaceae family through horizontal gene transfer. CHASRI underwent sequential assembly of two gene modules: cus (copper-sensing copper efflux system) and pco (plasmid-encoded copper resistance system), facilitating its dissemination. The presence of CHASRI alongside tetracycline and sulfonamide-macrolide resistance genes on a single plasmid suggests a significant evolutionary advantage in agricultural settings. In food production systems, heavy metals such as copper are frequently utilized as supra-nutritional feed additives for growth promotion, while silver is increasingly employed in antimicrobial applications, including facility disinfection and as emerging nano-based growth enhancers [21,22]. This environmental pressure can drive the persistence of multidrug-resistant (MDR) plasmids even in the absence of direct antibiotic use [23]. Through a process of co-selection, the exposure to heavy metals selects for the metal resistance island, which indirectly maintains the linked AMR genes within the bacterial population. This ‘hitchhiking’ mechanism ensures that the plasmid remains stable, effectively creating a reservoir of antibiotic resistance that persists despite reduced antibiotic consumption.

A study by the US-FDA investigated the prevalence of CHASRI in *Salmonella*, conducting phylogenetic clustering analysis along with source and serotype statistics on 4954 CHASRI-containing *Salmonella* deposited in the NCBI database [24]. The study identified poultry, pork, and environmental sources as the primary origins of CHASRI-containing *Salmonella*. CHASRI exhibits diverse forms in *Salmonella*. One identified form is a chromosomal mobile genetic island known as SGI-3, found in *Salmonella* 4 [5], 12:i:–. SGI-3 contains a CHASRI, in addition to an arsenic resistance operon, and genes associated with conjugative transfer and DNA replication or partitioning [25]. CHASRI was also found to be present in the large genomic island denoted as SGI-4, identified in *Salmonella enterica* serovar Monophasi [26]. The CHASRI identified in this study represented a previously unreported form in *Salmonella*. It exists within a multidrug-resistant plasmid alongside a tetracycline resistance gene cluster and a sulfonamide-macrolide-mercury resistance gene cluster. This co-occurrence suggests that the integration of CHASRI into this plasmid may have been driven by complex co-selection and cross-selection pressures during its evolutionary history.

### 3.4. Limitations of the Study

Several limitations of the current study should be acknowledged. Primarily, the *Salmonella* isolates analyzed were recovered between 2002 and 2009. While this historical collection serves as a crucial baseline to track the long-term persistence and evolutionary origins of complex MDR plasmids, such as pCFS0255-1, it may not accurately reflect the current epidemiological landscape, circulating serotypes, or contemporary AMR profiles of foodborne *Salmonella* in Colombia. Furthermore, the collection comprises a relatively modest sample size (n = 90), geographically restricted to specific departments in northern Colombia (Montería, Cartagena, Sincelejo, and Barranquilla), which may limit the broader generalizability of the findings across the entire country. Finally, although our sampling spans various food sources and food-producing animals within the One Health continuum, the absence of corresponding human clinical isolates from the same timeframe restricts the ability to definitively reconstruct the transmission dynamics of these resistant lineages from the food chain to human infections. Despite these limitations, the genomic characterization presented here offers valuable insights into the historical AMR phenotypes and genotypes and reinforces the necessity of implementing continuous, centralized WGS-based surveillance to track emerging MDR threats.

## 4. Materials and Methods

### 4.1. Bacterial Isolates

A collection of 90 *Salmonella* species recovered between 2002 and 2009 from a variety of food products and food-producing animals in Colombia was obtained from the University of Cordoba (Colombia). All bacterial isolates were streaked on XLD medium (Oxoid, Basingstoke, England) to check for purity and confirmed as *Salmonella* using a *Salmonella* latex test (Oxoid). These were then stored at −80 °C in glycerol stocks. The serotype data for laboratory testing were provided by the University of Cordoba (Colombia). Metadata including sources, years, and locations of all isolates and the corresponding lab-tested serotypes are shown in Appendix A Table A1.

### 4.2. Antibiotic Susceptibility Testing (AST)

Susceptibilities to 15 antimicrobial compounds were determined by disk diffusion and interpreted according to the Clinical and Laboratory Standards Institute (CLSI) guidelines (2007) [27]. *Escherichia coli* ATCC^®^ 25922 (American Type Culture Collection, Manassas, VA, USA) was included as a control.

Minimum inhibitory concentrations (MIC) for nalidixic acid (Sigma-Aldrich, Arklow, Ireland) and ciprofloxacin (Sigma-Aldrich) were determined by broth microdilution, in the absence and presence of 40 µg/mL phenyl-arginine-ß-naphthylamide (PAßN) (Sigma-Aldrich), to assess the presence of active efflux mechanisms.

### 4.3. Whole-Genome Sequencing and Data Analysis

Short-read sequences were obtained using Illumina HiSeqX platform (Illumina, San Diego, CA, USA) and the 150 bp paired-end approach, from libraries with an average insert size of 350 bp. Raw sequences were initially trimmed using Trimmomatic (v0.39) [28] followed by de novo assembly for complete genomes performed using SPAdes (v3.15.3) [29]. A subset of the isolate collection was re-sequenced using long-read sequencing methods on a MinION platform. Assembly was performed using Canu (v2.2) with hybrid assembly method to NGS reads [30] and sequences were polished using NextPolish (v1.4.1-0) [31]. *Salmonella* genome assemblies were annotated with the prokaryotic genome annotation tool Prokka (v1.14.6) [32]. Antibiotic resistance genes were predicted using the ABRicate (v1.0.0) software package, combining three reference databases, CARD (v3.2.9) [33], ResFinder (v4.5.0) [34], and NCBI AMRFinderPlus (v2024-05-02.2) [35]. Gene names were unified to the NCBI AMRFinderPlus references and all resistance genes were screened using the BLASTN algorithm, with minimum nucleotide identity and alignment length coverage of 80%.

Plasmids markers were predicted using PlasmidFinder (v2.1.6) [36], and the sequences were validated using BLAST (BLASTN). Sequence hits against reference sequences with the best coverage and identity scores were recorded. Since any single reference sequence could not completely cover the plasmids found in this study, partially covered reference sequences with high identity were also selected, including a transposon sequence, to ensure complete coverage of the plasmids in this study. Other mobile genetic elements were annotated using MGEFinder (v1.0.6) [37]. Sequence structure and genes of each plasmid were visualized using Brig (v0.95) and compared against reference sequences using BLASTN [38]. Partial sequence comparisons for the MDR gene islands and up-/down-streams were visualized using EasyFig (version 2.2.3) [39].

Serotypes of these isolates were predicted in silico from WGS assemblies using SeqSero2 (version 1.0.1) [40], and multi-locus sequence typing (MLST) of the isolates was predicted with reference to the PubMLST database using the MLST tool (v 2.20.0) [41]. The *Salmonella* pathogenicity islands (SPIs) were identified by PAIDB (v2.0), using the BLASTN method [42], and a threshold and coverage percentage of 90 was used for comparison with the reference sequences.

Core genomes were calculated using Roary (v3.13.0) from all 90 WGS assemblies [43]. Snippy (v4.6.0) was applied to clean up core genome alignment [44]. Gubbins (v3.2.1) and SNP-sites (v2.3.1) were used to filter the single nucleotide polymorphism alignment from the cleaned core genome alignment [45,46]. A maximum-likelihood phylogenetic tree based on the extracted core-genome SNPs was constructed using FastTree (v2.1.10) with the JC + CAT model, and the root of the tree was adjusted to its midpoint [47]. The final outputs were used for the illustration of tree and performed using Biopython (version 1.79).

## 5. Conclusions

In conclusion, this study developed a whole-genome analysis workflow based on core-genome SNPs for monitoring global foodborne pathogen transmission risks. This workflow was applied to assess and predict the resistance and pathogenicity of 90 *Salmonella* from Colombia. The study bacterial samples were identified by these methods to be of different serotypes, capable of predicting AMR-encoding genes and SPIs. A multidrug-resistant isolate CFS0255, characterized by two resistance-encoding plasmids, was described using a combination of short- and long-read sequencing. Furthermore, the emergence of plasmid pCFS0255-1 was hypothesized based on a multi-stepped integration process involving three resistance gene clusters, including: the TET operon, a CHASRI, and the SUL-MEF-MER cluster.

## Figures and Tables

**Table 1:** Reference sequences for AMR gene regions in WGS assemblies.

Strain Name	Accession # *	Genes	Ref Start	Ref End
*Escherichia coli* strain FDAARGOS_1285 chromosome	CP070152.1	*sul2*	3,298,314	3,314,284
*Salmonella enterica* subsp. *enterica* serovar Agona strain CVM N18S0017 chromosome	CP082615.1	*sul2*	4,494,856	4,510,826
*Escherichia coli* plasmid pECY6-7	GQ374156.1	*qnrB19*	1	2699
*Salmonella enterica*, complete plasmid pSGI15	FN428572.1	*qnrB19*	1	2699
*Salmonella enterica* subsp. *enterica* serovar Typhi strain 311189_217186 plasmid pHCM1	CP029645.1	*tetB*, *(tetC*, *tetR)*	164,340	170,822
*Escherichia coli* strain O177:H21 chromosome	CP016546.1	*aph(3″)Ib*, *aph(6)Id*, *aph(3′)Ia*	3,227,380	3,242,186
*Shigella sonnei* Ss046 plasmid pSS046_spA	CP000641.1	*tetA*, *sul2*, *aph(3″)Ib*, *aph(6)Id*, *(tetR)*	1	8401
*Escherichia coli* strain ECwhn14 plasmid p8401	CP012198.1	*tetA*, *sul2*, *aph(3″)Ib*, *aph(6)Id*, *(tetR)*	1	8401
*Salmonella enterica* subsp. *enterica* serovar *Schwarzengrund* strain CVM N18S1602 plasmid pN18S1602-2	CP082555.1	*tetA*, *sul2*, *aph(3″)Ib*, *(tetR)*	1	9473
*Shigella flexneri* 1c strain Y394 plasmid pNV-Y394	CP030774.1	*tetA*, *sul2*, *aph(3″)Ib*, *(tetR)*	1	10,866
*Escherichia coli* strain WW16 IncQ1 plasmid pQGU16	MH718732.1	*tetA*, *sul2*, *aph(3″)Ib*, *(tetR)*	1	14,146

* The Accession number of corresponding reference sequences in the NCBI database.

**Table 2:** PlasmidFinder results for CFS0255 complete genome.

Database	Plasmid	Identity%	Query/Template Length	Contig	Position in Contig	Accession Number
Enterobacteriales	IncFIA(HI1)	100	388/388	contig_4	206,302–206,689	AF250878
Enterobacteriales	IncHI1A	100	420/420	contig_4	50,119–50,538	AF250878
Enterobacteriales	IncHI1B(R27)	100	540/540	contig_4	63,614–64,153	AF250878
Enterobacteriales	IncI1-I(Alpha)	100	142/142	contig_5	53,314–53,455	AP005147

## Data Availability

Metadata of the strains used in this study is listed in Appendix A. WGS data is available upon request.

## Supplementary Materials

The following supporting information can be downloaded at: https://www.mdpi.com/article/10.3390/antibiotics15050511/s1, Supplementary Table S1: BLASTN search results and metadata of highly similar plasmids to pCFS0255-1.

## Abbreviations

The following abbreviations are used in this manuscript:

AMR	Antimicrobial Resistance
ANI	Average Nucleotide Identity
AST	Antimicrobial Susceptibility Testing
CARD	Comprehensive Antibiotic Resistance Database
CHASRI	Copper Homeostasis and Silver Resistance Island
CLSI	Clinical and Laboratory Standards Institute
COIPARS	Colombian Integrated Program for Antimicrobial Resistance Surveillance
HGT	Horizontal Gene Transfer
Inc	Incompatibility Group
IS	Insertion Sequence
MDR	Multidrug Resistant/Multidrug Resistance
MGE	Mobile Genetic Element
MIC	Minimum Inhibitory Concentration
MLST	Multi-Locus Sequence Typing
NGS	Next-Generation Sequencing
NTS	Non-Typhoidal *Salmonella*
PAßN	Phenyl-Arginine-ß-Naphthylamide
SNP	Single Nucleotide Polymorphism
SPI	*Salmonella* Pathogenicity Island
ST	Sequence Type
WGS	Whole-Genome Sequencing
XLD	Xylose Lysine Deoxycholate

## Appendix A

**Table A1:** Metadata of isolates in this study.

Strain Name	Year	Source	Area/Department	Lab-Tested Serotype
CFS0201	2002	ham	Barranquilla	Carrau
CFS0202	2003	ham	Barranquilla	Carrau
CFS0203	2004	cheese	Sincelejo	Anatum
CFS0204	2004	chicken	Sincelejo	Infantis
CFS0205	2004	cheese	Sincelejo	Infantis
CFS0206	2004	meat	Sincelejo	Newport
CFS0207	2004	chicken	Sincelejo	Newport
CFS0208	2004	sausages	Sincelejo	Newport
CFS0209	2004	cheese	Sincelejo	Infantis
CFS0210	2004	chicken	Cartagena	Uganda
CFS0211	2004	chicken	Cartagena	Uganda
CFS0212	2004	meat	Cartagena	Uganda
CFS0213	2004	chicken	Cartagena	Uganda
CFS0214	2004	meat	Cartagena	Uganda
CFS0215	2004	ground meat	Sincelejo	Newport
CFS0216	2004	ham	Montería	Newport
CFS0217	2004	ham	Montería	Newport
CFS0218	2004	chicken	Montería	Newport
CFS0219	2004	chicken	Montería	Braenderup
CFS0220	2004	chicken	Sincelejo	Infantis
CFS0221	2005	ham	Barranquilla	Minnesota
CFS0222	2005	intestine bovine	Barranquilla	Adelaide
CFS0223	2005	salty meat	Barranquilla	Newport
CFS0224	2005	sausages	Monteria	Uganda
CFS0225	2005	pig	Monteria	Newport
CFS0226	2005	chicken	Monteria	Infantis
CFS0227	2005	ham	Monteria	Anatum
CFS0228	2005	cheese	Cartagena	Anatum
CFS0229	2005	meat	Cartagena	Uganda
CFS0230	2005	intestine bovine	Cartagena	Braenderup
CFS0231	2005	meat	Cartagena	Saintpaul
CFS0232	2005	Bovine spleen	Cartagena	Saintpaul
CFS0233	2005	sausages	Monteria	Anatum
CFS0234	2005	ground meat	Monteria	Rubislaw
CFS0235	2005	ground meat	Monteria	Rubislaw
CFS0236	2005	mixture corn and egg	Monteria	Rubislaw
CFS0237	2005	potato	Cartagena	Muenchen
CFS0238	2005	ground meat	Cartagena	Uganda
CFS0239	2005	ground meat	Cartagena	Uganda
CFS0240	2005	meat	Cartagena	Uganda
CFS0241	2005	sausages	Cartagena	Carrau
CFS0242	2005	*Amazona* spp.	Monteria	Anatum
CFS0243	2005	cheese	Cartagena	Anatum
CFS0244	2005	ground meat	Cartagena	Anatum
CFS0245	2005	meat	Cartagena	Carrau
CFS0246	2005	cheese	Cartagena	Carrau
CFS0247	2005	ground meat	Cartagena	Uganda
CFS0248	2007	sausages	Sincelejo	Anatum
CFS0249	2007	sausages	Sincelejo	Saintpaul
CFS0250	2007	cheese	Sincelejo	Anatum
CFS0251	2007	cheese	Sincelejo	Carrau
CFS0252	2005	ground meat	Cartagena	Carrau
CFS0253	2005	ham	Cartagena	Anatum
CFS0254	2005	cheese	Cartagena	Anatum
CFS0255	2005	sausages	Cartagena	Anatum
CFS0256	2005	ground meat	Cartagena	Anatum
CFS0257	2005	ham	Cartagena	Anatum
CFS0258	2005	cheese	Cartagena	Muenchen
CFS0259	2005	sausages	Cartagena	Uganda
CFS0260	2005	meat	Cartagena	Saintpaul
CFS0261	2005	cheese	Cartagena	Muenchen
CFS0262	2006	ham	Monteria	Braenderup
CFS0263	2006	ground meat	Monteria	Braenderup
CFS0264	2006	cheese	Monteria	Muenchen
CFS0265	2007	salty meat	Monteria	Uganda
CFS0266	2007	salty meat	Monteria	Uganda
CFS0267	2007	ham	Monteria	Derby
CFS0268	2007	sausages	Monteria	Uganda
CFS0269	2007	sausages	Monteria	Braenderup
CFS0270	2007	sausages	Monteria	Uganda
CFS0271	2007	flour mandioka powder	Monteria	Braenderup
CFS0272	2007	flour mandioka powder	Monteria	Uganda
CFS0273	2007	ground meat	Monteria	Uganda
CFS0274	2007	meat	Monteria	Bredeney
CFS0275	2007	ground meat	Monteria	enterica subsp. enterica ser. 6,7:d:-
CFS0276	2007	cheese	Monteria	Senftenberg
CFS0277	2007	cheese	Monteria	Braenderup
CFS0278	2007	ground meat	Monteria	Saintpaul
CFS0279	2007	meat	Monteria	Braenderup
CFS0280	2007	ground meat	Monteria	Uganda
CFS0281	2007	ground meat	Monteria	Senftenberg
CFS0282	2007	sausages	Monteria	Braenderup
CFS0283	2007	sausages	Monteria	Javiana
CFS0284	2007	sausages	Monteria	Fresno
CFS0286	2007	ground meat	Monteria	Gaminara
CFS0287	2007	potato and meat	Monteria	Infantis
CFS0288	2007	*Iguana Iguana*	Monteria	Braenderup
CFS0289	2009	*Hydrochoerus hydrochaeris*	Monteria	Fresno
CFS0290	2009	*Hydrochoerus hydrochaeris*	Monteria	Typhimurium
CFS0291	2009	*Trachemys scripta callirostris*	Monteria	Javiana
