# Supplementary material for: Genomic Epidemiology of Foodborne Salmonella in Colombia (2002–2009): Emergence of Novel IncHI1 and IncI1 Plasmids Harboring Metal and Multi-Drug Resistance Clusters
Source: Antibiotics (Basel). 2026 May 18;15(5):511. doi: 10.3390/antibiotics15050511 (PMC13203441; doi:10.3390/antibiotics15050511)
Supplement: Supplementary file 1 [file antibiotics-15-00511-s001.zip › antibiotics-4201072-supplementary.pdf]

Supplementary Table S1. BLASTN search results and metadata of highly similar plasmids to pCFS0255-1

Subject ID	Sequence Description	Weighted ANI (%)	Query Coverage (%)	Species	Collection Date	Location	Source
CP123651.1	Salmonella enterica strain 19MN02PC03 plasmid pN19S0223-1, complete sequence	97.346	95.49	*Salmonella enterica*	2019	USA	pork chop
CP064017.1	Escherichia coli strain EC71PP1 plasmid p71PP1-IncHI1-mcr-3.1, complete sequence	97.174	89.958	*Escherichia coli*	2018	Thailand	rectal swab
CP082572.1	Salmonella enterica subsp. enterica serovar 4,[5],12:i:- strain CVM N18S0993 plasmid pN18S0993-1, complete sequence	97.187	89.054	*Salmonella enterica*	2018	USA	ground turkey
CP053049.1	Salmonella enterica subsp. enterica serovar Derby strain CVM 30155 plasmid p30155-1, complete sequence	97.241	87.712	*Salmonella enterica subsp. enterica serovar Derby*	2003	USA	swine
CP022495.1	Salmonella enterica subsp. enterica serovar Derby strain SA20035215 plasmid unnamed1, complete sequence	97.396	87.687	*Salmonella enterica subsp. enterica serovar Derby*	unknown	unknown	unknown
LT795503.1	Escherichia coli strain KV7 genome assembly, plasmid: I	97.366	87.394	*Escherichia coli*	unknown	unknown	pig feces
CP060850.1	Salmonella enterica strain 724 plasmid p724-MDR, complete sequence	97.274	84.978	*Salmonella enterica*	2019	China	feces
CP060835.1	Salmonella enterica strain 1505 plasmid p1505-MDR, complete sequence	96.824	84.978	*Salmonella enterica*	2015	China	feces

CP091605.1	Salmonella enterica strain 1902 plasmid p1902-MDR, complete sequence	96.816	84.978	*Salmonella enterica*	2021	China	feces
CP060847.1	Salmonella enterica strain 733 plasmid p733-MDR, complete sequence	96.813	84.978	*Salmonella enterica*	2019	China	feces
CP060838.1	Salmonella enterica strain 852 plasmid p852-MDR, complete sequence	96.8	84.978	*Salmonella enterica*	2019	China	feces
CP060841.1	Salmonella enterica strain 805 plasmid p805-MDR, complete sequence	96.8	84.978	*Salmonella enterica*	2019	China	feces
CP060845.1	Salmonella enterica strain 770 plasmid p770-MDR, complete sequence	96.8	84.978	*Salmonella enterica*	2019	China	feces
CP065568.1	Salmonella enterica subsp. enterica serovar Typhimurium strain R18.1078 plasmid pR18.1078_p247k, complete sequence	96.8	84.978	*Salmonella enterica subsp. enterica serovar Typhimurium*	2018	China	stool
CP091616.1	Salmonella enterica strain 1653 plasmid p1653-MDR, complete sequence	96.8	84.978	*Salmonella enterica*	2021	China	feces
CP091627.1	Salmonella enterica strain 1165 plasmid p1165-MDR, complete sequence	96.8	84.978	*Salmonella enterica*	2020	China	feces
CP091631.1	Salmonella enterica strain 1147 plasmid p1147-MDR, complete sequence	96.8	84.978	*Salmonella enterica*	2020	China	feces
CP060586.1	Salmonella enterica strain GX1006 plasmid pSal21GXH-tetX4, complete sequence	96.796	84.978	*Salmonella enterica*	2016	China	stool

MG874042.1	Salmonella sp. strain Sa4 plasmid pSa4-CIP, complete sequence	96.791	84.978	*Salmonella sp.*	2014	unknown	meat
CP149336.1	Salmonella enterica subsp. enterica serovar Typhimurium strain Z1323SSL0010 plasmid pZ1323SSL0010-2, complete sequence	97.28	84.977	*Salmonella enterica subsp. enterica serovar Typhimurium*	2023	South Korea	unknown
CP060853.1	Salmonella enterica strain 467 plasmid p467-MDR, complete sequence	97.593	84.668	*Salmonella enterica*	2018	China	feces
AP020333.1	Salmonella enterica SESen3709 plasmid pSESen3709_1 DNA, complete genome	96.771	84.264	*Salmonella enterica*	2017	Japan	feces
CP068510.1	Salmonella enterica subsp. enterica serovar Derby strain 19CS0402 plasmid pIncH1, complete sequence	96.82	84.245	*Salmonella enterica subsp. enterica serovar Derby*	2013	Viet Nam	pork at market
KX129784.1	Escherichia coli strain H226B plasmid pH226B, complete sequence	97.732	83.509	*Escherichia coli*	unknown	unknown	unknown
CP072461.1	Klebsiella pneumoniae strain K-1L plasmid pK-1L-1, complete sequence	96.72	82.698	*Klebsiella pneumoniae*	2019	China	unknown
CP091608.1	Salmonella enterica strain 1810 plasmid p1810-MDR, complete sequence	96.745	82.403	*Salmonella enterica*	2021	China	feces
CP030182.1	Salmonella enterica strain SA20030575 plasmid pSA20030575.1, complete sequence	97.91	81.858	*Salmonella enterica*	unknown	unknown	unknown
CP139177.1	Escherichia coli strain JX21CE33 chromosome, complete genome	97.261	81.726	*Escherichia coli*	2021	China	unknown
